# Effect of maternal mental health improvement programs on obesity in pediatric populations: a protocol for a systematic review and meta-analysis

**DOI:** 10.1186/s13643-018-0798-2

**Published:** 2018-08-29

**Authors:** Abdul Wajid, Muhammad Kashif Mughal, Deborah McNeil, Helen Lee Robertson, Dawn Kingston

**Affiliations:** 1 University of Calgary, PF 2220, 2500 University Dr. NW, Calgary, AB T2N 1N4 Canada; 2 Alberta Health Services, Southport Atrium, #2237, 10101 Southport Rd, SW, Calgary, AB T2W 3N2 Canada

**Keywords:** Maternal mental health, Childhood obesity, Systematic review, Meta-analysis, Protocol

## Abstract

**Background:**

Childhood obesity has become a global epidemic irrespective of the socioeconomic status of a country or nation. Obesity increases the risk of various diseases in children, for example asthma, sleep apnea, bone and joint problems, type-2 diabetes, and heart problems. The existing literature informs us of the many factors associated with childhood obesity. Among these factors, maternal mental health has been found to be a strong predictor. Maternal mental health programs were implemented to address the issue of childhood obesity but with little or no improvement. It suggests systematically reviewing the literature to assess the contents of these programs and carrying out meta-analysis for the overall effect of these interventions.

**Methods:**

The studies included in this review will be experimental designs such as randomized controlled trials (RCTs) which provide information on interventions to improve maternal mental health and its effects on childhood obesity. We plan to search MEDLINE, EMBASE, Cochrane Central Register of Controlled Trials, PsycINFO, ERIC, CINAHL, ProQuest Dissertations and Theses Global, Scopus, and Web of Science with no restrictions as to language. Reference lists of the selected articles will also be searched for additional articles. The Cochrane EPOC Risk of Bias Tool will be used to assess the quality of studies. If the studies lend themselves to a statistical analysis, we will also carry out a meta-analysis.

**Discussion:**

This review will help determine the effect of maternal health improvement programs on childhood obesity. These findings, in turn, will guide the research community on the development of related programs in the future.

**Systematic review registration:**

PROSPERO: CRD42017072737.

**Electronic supplementary material:**

The online version of this article (10.1186/s13643-018-0798-2) contains supplementary material, which is available to authorized users.

## Background

For about three decades, childhood and adolescent obesity estimates have been at alarming levels [1]. In 2016, estimates show that more than 40 million children under 5 years of age across the globe are either overweight or obese [1–3]. In addition, around 340 million individuals up to the age of 18 years are overweight or obese. More than 85% of these children are from high-income countries. This suggests that approximately as high as one-third of the children in high-income countries and around one-tenth in middle and low-income countries are either overweight or obese [4].

Overweight and/or obese children and adolescents are more likely to develop asthma, sleep apnea, bone and joint problems, type-2 diabetes, and heart problems [5–7]. Childhood obesity is associated with adverse consequences on the cardiovascular system suggesting initiation of atherosclerotic changes in vessels in early life [8, 9], a higher risk of high blood pressure [10, 11] and about seven times higher risk of triglycerides in 5–10-year-old obese children as compared to non-obese [12, 13]. These children are also more likely to experience social and psychological problems such as bullying, social isolation, and low self-esteem as compared to their normal-weight peers [14].

Importantly, outcomes of childhood obesity are not only limited to childhood. Obese children are approximately 17 times more likely to become obese during adulthood. They are more likely to grow up as obese adults and develop chronic diseases such as cardiovascular diseases and diabetes comparatively at an early stage [15, 16], with higher incidence of non-communicable diseases such as type-2 diabetes, heart problems, metabolic syndrome, and certain types of cancers at a younger age [17–19].

There is a wide range of risk factors which start from the antenatal period until late childhood. Moreover, risk factors also affect others, such as the mother or father in terms of their physical or mental health, and their behaviors related to smoking, drugs, and alcohol. Antenatal depression and depression are also associated with a 2–3-fold increased risk of childhood obesity [20–22].

Literature suggests that maternal factors play a significant role in the development of childhood overweight and obesity. These factors could be pre-pregnancy BMI or gestational weight gain physical activity or smoking, or maternal physical and mental health [23–26]. Among these factors, maternal mental health influences the lifestyle which puts a child at a higher risk of being overweight or obese. Prenatal mental problems may impact the care of self and meet the needs of the pregnancy. Similarly, postnatal depression depending upon the age of the newborn may affect breastfeeding, hygiene and cleanliness, timing and contents of initiation solids, physical activity of the child and screen time, and use of junk food. Childhood obesity is not a result of one or two factors but results from a multitude of causes. For optimum results, it is not wrong to address the issue of overweight and obesity in this age group by designing and implementing interventions for as many factors as possible, including maternal mental health.

Maternal mental health issues may exist any time from the antenatal period until late childhood to adolescent, potentially leading a child toward overweight and obesity. There is a dearth of evidence on the assessment of mental health interventions in relation to weight or adiposity in pediatric age groups. To our knowledge, there are three systematic reviews conducted on childhood obesity treatment and interventions [27–29]. None of these reviews had a study on maternal mental health. Staniford et al. identified articles on cognitive behavioral therapy (CBT), but therapy was related to coaching and motivation for goal setting and becoming more disciplined toward diet and physical activity [27]. Literature shows the role of maternal depression and distress in childhood obesity without any systematic assessment of related interventions. It seems high time to design and conduct a systematic review for assessing the role of maternal mental health in addressing the issue of pediatric obesity. The findings will guide us as to where to focus as far as mental health is concerned.

### Objectives

The objective of this systematic review and meta-analysis is to evaluate the effectiveness of the programs implemented to prevent childhood obesity by improving maternal mental health.

## Methods

### Protocol and registration

We will use the preferred reporting items for systematic reviews and meta-analyses protocols (PRISMA-P) guideline for reporting this protocol and follow Cochrane methods for carrying out the planned systematic review. For this purpose, the PRISMA-P checklist is provided (Additional file 1).

### Eligibility criteria

Studies will be selected following the criteria stated below:

#### Population

Pregnant and postpartum women with their children up to the age of 21 years.

#### Intervention

All interventions intending to control/prevent maternal mental health issues will be selected. The intervention could include counseling, psychotherapy, peer support, and other related areas directed at improving maternal mental health during pregnancy. Modes of intervention include face-to-face, online, support group activities, yoga, exercise, socialization, or volunteer work with intervention follow up for up to a period of 15 years. There will be no restrictions on studies by the site of research or intervention.

There will be no restrictions on the language of published articles, ethnicity, and parity. We will not exclude studies based on the measurement (self-reported vs measured). If needed, we can conduct sub-group analysis. We will attempt to obtain translations of the key sections of non-English language studies in order to complete the review process. For translation purpose, we will utilize the Cochrane Task Exchange service [30]. However, a list of titles of related studies will be provided in case translation is not accomplished.

#### Comparison

Usual care/no intervention or an intervention that does not contain a maternal mental health component.

#### Outcome

##### Primary outcome

Infant/child overweight and obesity will be assessed by percentile if they are under 2 years or BMI if over 2 years [31]  as the primary outcome. In case, BMI is documented as BMI *z*-score or BMI-SDS; we will capture that as well.

##### Secondary outcome

We will also look for any other measures used for the assessment of adiposity, such as skin-fold thickness or a hip-waist ratio.

#### Exclusion criteria

We will not include quasi-experimental design and any other interventions where recruitment does not utilize randomization. Any co-morbidities, such as diabetes mellitus, or participants with other endocrinologic diseases will also be excluded.

#### Type of Studies

We will include studies with experimental design, for example randomized controlled trials (RCTs), clinical trials, and cluster RCTs (CRCTs).

PROSPERO registration no: This protocol is registered with PROSPERO with ID CRD42017072737.

### Search strategy

A preliminary search strategy (Additional file 2) was developed and refined by one of the investigators (AW) and a medical librarian (HLR). Citations were found by searching the following databases from the first date available: Epub Ahead of Print, In-Process & Other Non-Indexed Citations, Ovid MEDLINE Daily and Ovid MEDLINE, EMBASE, Cochrane Central Register of Controlled Trials, PsycINFO, ERIC, CINAHL, ProQuest Dissertations and Theses Global, Scopus, and Web of Science. Gray literature sources that will be searched include clinicaltrials.gov, ANZCTR, WHO International Clinical Trials Registry Platform, AHRQ, and OpenGrey, combinations of subject headings, keywords, and synonyms used include childhood obesity, pregnancy, mothers, maternal mental health, anxiety disorders, panic disorder, depression, psychological stress, and bipolar disorder. The trials published after 1990 will be included as scoping search could not find related trials before 1990 [29]. Reference lists of included studies will be hand-searched. We will correspond with authors to clarify study procedures, if necessary.

### Study records

#### Data management

The search result data will be transferred to EndNote X8 for the removal of duplicate records and retrieval of articles.

#### Selection process

Two review authors (AW and KM) will independently screen the titles and abstracts obtained from the search for key words related to BMI and childhood obesity. Full text of potentially relevant articles will be reviewed for study inclusion. Eligibility disagreements will be resolved by consensus. If consensus cannot be reached, final decisions will be made by a third independent reviewer in the team. Reasons for exclusion will be documented.

#### Data collection process

Data collection: A data extraction form will be developed and at least 5% will be pilot-tested for this review. Two reviewers (AW) and (KM) will independently carry out data extraction.

### Data items

#### List of variables

We will document key variables including the intervention, which will include, but not be limited to, year of publication, the content of interventions, type of interventions (psychotherapy and pharmacotherapy), duration of symptoms, the severity of symptoms, compliance, blinding, and data management and analysis. Other data include participants’ characteristics, type of comparison groups, trial size and type, and duration of follow up. The findings will be documented in tabulated form.

#### List of outcomes

Under 2 years, overweight and obesity by percentile, and over 2 years of age BMI will be assessed. Depending upon the documentation of BMI variable, we will assess as BMI *z*-score or cut-off for overweight and obesity. We will also look for any other measures used for the assessment of adiposity, such as skin-fold thickness or waist-to-hip ratio.

#### Risk of bias assessment

The Cochrane EPOC Risk of Bias Tool for RCTs will be used to assess the risk of bias or quality of individual studies. This tool grades the studies at low, high, or unclear risk of bias [32]. According to this tool, domains will be addressed: random sequence generation bias, allocation concealment bias, selective reporting bias, performance bias, detection bias, attrition bias, and other bias. These are further categorized as the low, high, or unclear risk of bias based on the type of responses. The task will be completed by two reviewers (AW and KM).

### Data synthesis

#### Qualitative synthesis

As the first step in data synthesis, we will carry out a qualitative analysis. As our exposure of maternal mental health expands up to adolescence, we will synthesize the data according to pediatric age groups, such as infants (up to 2 years), preschoolers (2–5 years), early childhood (5–8 years), late childhood (8–12 years), and adolescents (12–18 years). Similarly, we will handle the data by number, type, and frequency of interventions. Depending upon the type of trials, we will synthesize data by trial type, for RCT and cluster RCTs.

#### Quantitative synthesis

We plan to pool the results if heterogeneity of the studies permits. Heterogeneity will be assessed by Chi^2^ statistics and I^2^ statistics. The first assesses the presence of heterogeneity as determined by *p* value (< .05) while the second quantifies it [33]. We will not pool the results if heterogeneity is substantial (50% or higher). We will use a random-effects model when pooling the results; this does not remove the heterogeneity but it considers the existence of heterogeneity while carrying out the pooling (heterogeneity and sub-group analysis).

#### Summary statistics and method of analysis

The odds ratio (OR) will be used as a measure of effect with its 95% CI, by using either random effect models as we expect heterogeneity in studies [34]. Depending upon the types of analysis, summary statistics may change; in that case, we will report and document those summary statistics and compare them with the similar statistics, such as risk ratio or risk difference.

We will follow Cohen’s d [35] for the assessment of effect size; eventually, a decision on effectiveness will be made.

#### Subgroup analyses

We will conduct sub-group analysis by type of intervention, study design, or children age group depending upon the articles included in the systematic review.

#### Additional analyses

We plan to carry out sensitivity analysis following the quality of the trial. We will compare the estimates of all trials with that of with high quality.

### Meta-bias

While bias in an individual study suggests some sort of procedural issue, in conducting a meta-analysis, bias is introduced as a meta-bias, including publication bias [36]. We will take measures to address this issue, including reviewing the clinical trials register for registered trials that have not been published after July 1, 2005 [37]. Furthermore, we will create funnel plots when a number of studies goes beyond 10 to assess reporting bias [38].

### Cumulative estimate

The Grading of Recommendations Assessment, Development, and Evaluation (GRADE) will be utilized to assess the quality of evidence. This will be graded as high, moderate, low, and very low. This will be independently accomplished by AW and KM. the results will be presented with a summary of finding a table. For downgrading, we will use risk of bias, inconsistency, indirectness, imprecision, and publication bias. Upgradation will be assessed by the large magnitude of the effect, dose-response effect, and effect of all plausible factors [39].

## Discussion

This protocol is the first in the series of our planned systematic reviews and meta-analyses. The findings of this systematic review and meta-analysis will inform the level of body evidence which will eventually guide us if more research is needed. At the same time, this review will provide us the strength of programs with a variety of intervention by contents and duration. All efforts will be made to capture as many related databases as possible, including theses and dissertations. The core purpose is to determine which intervention package was effective to what extent. To make best use of the outcome, we will disseminate findings to the related audience such as academia, clinicians, and communities in addition to the peer-reviewed publications.

## Additional files

Additional file 1: PRISMA-P Checklist. (DOCX 20 kb)

Additional file 2: Search strategy MEDLINE. (DOCX 15 kb)

## Abbreviations

BMI: Body mass index
GRADE: The Grading of Recommendations Assessment, Development, and Evaluation (GRADE)
OR: Odds ratio
PRISMA-P: The Preferred reporting items in systematic reviews and meta-analyses for protocols
PROSPERO: International prospective register of systematic reviews
RCT: Randomized controlled trial
