# Supplementary material for: Effect of maternal mental health improvement programs on obesity in pediatric populations: a protocol for a systematic review and meta-analysis
Source: Syst Rev. 2018 Aug 29;7:133. doi: 10.1186/s13643-018-0798-2 (PMC6116373; doi:10.1186/s13643-018-0798-2)
Supplement: Supplementary file 2 — Search strategy MEDLINE. (DOCX 15 kb) [file 13643_2018_798_MOESM2_ESM.docx]

**Additional file 2**

Search strategy for MEDLINE

1. Pediatric Obesity/
2. ((pediatric* or paediatric* or child* or adolescen* or preschool or pre-school or infant* or infanc* or toddler*) adj3 (obesity or obese or overweight or over-weight or body mass index or bmi or adiposity)).mp.
3. or/1-2
4. Mothers/
5. Pregnancy/
6. (mothers or maternal).mp.
7. (pregnanc* or pregnant or prenatal or pre-natal or antenatal or ante-natal or expectant).mp.
8. (postnatal or post-natal).mp.

9. or/4-8

10. ((maternal or mother or mothers) adj2 (mental health or psychopatholog*)).mp.

11. anxiety disorders/

12. Obsessive-Compulsive Disorder/

13. Panic Disorder/

14. Depression/

15. exp Depressive Disorder/

16. Stress, Psychological/

17. Bipolar Disorder/

18. (anxiety or panic or depression* or depressive or dysthymia or dysthymic or bipolar or manic-depress* or obsessive compulsive).ti,ab,kf.

19. (stress adj5 (mental or psycholog* or psychic or disorder*)).ti,ab,kf.

20. or/10-19

21. 3 and 9 and 20
